# Revealing the Multi-Target Mechanisms of Fespixon Cream in Diabetic Foot Ulcer Healing: Integrated Network Pharmacology, Molecular Docking, and Clinical RT-qPCR Validation

**DOI:** 10.3390/cimb47070485

**Published:** 2025-06-25

**Authors:** Tianbo Li, Dehua Wei, Jiangning Wang, Lei Gao

**Affiliations:** Orthopedic Department, Capital Medical University Affiliated Beijing Shijitan Hospital, No.10 Yangfangdian Tieyi Road, Haidian District, Beijing 100038, China; litianbo3285@bjsjth.cn (T.L.); wdh18404906316@163.com (D.W.); wangjn@bjsjth.cn (J.W.)

**Keywords:** diabetic foot ulcer, network pharmacology, molecular docking, Fespixon cream, multi-target mechanism

## Abstract

**Objective:** This study aims to elucidate the potential mechanisms by which Fespixon cream promotes diabetic foot ulcer (DFU) healing using network pharmacology, molecular docking, and RT-qPCR validation in clinical tissue samples. **Methods:** Active components of Fespixon cream were screened from the Traditional Chinese Medicine Systems Pharmacology Database (TCMSP) and relevant literature, and their corresponding targets were standardized using the Universal Protein Resource (UniProt) database. Diabetic foot ulcer (DFU)-related targets were retrieved and filtered from the GeneCards database and the Online Mendelian Inheritance in Man (OMIM) database. The intersection of drug and disease targets was identified, and a protein–protein interaction (PPI) network was constructed using the Search Tool for the Retrieval of Interacting Genes/Proteins (STRING) database. The interaction network was visualized using Cytoscape version 3.7.2 software. The potential mechanisms of the shared targets were analyzed by Gene Ontology (GO) and Kyoto Encyclopedia of Genes and Genomes (KEGG) pathway enrichment analysis using R software packages, and results were visualized through Bioinformatics online tools. Molecular docking was performed to validate the binding between key active compounds of Fespixon cream and core DFU targets using AutoDock Vina version 1.1.2 and PyMOL software. Furthermore, RT-qPCR analysis was performed on wound edge tissue samples from DFU patients treated with Fespixon cream to experimentally verify the mRNA expression levels of predicted hub genes. **Results:** Network pharmacology analysis identified eight active compounds in Fespixon cream, along with 153 potential therapeutic targets related to diabetic foot ulcer (DFU). Among these, 21 were determined as core targets, with the top five ranked by degree value being RAC-αserine/threonine-protein kinase (AKT1), Cellular tumor antigen p53 (TP53), Tumor necrosis factor (TNF), Interleukin-6 (IL6), and Mitogen-activated protein kinase 1 (MAPK1). GO enrichment analysis indicated that the targets of Fespixon cream were primarily involved in various biological processes related to cellular stress responses. KEGG pathway enrichment revealed that these targets were significantly enriched in pathways associated with diabetic complications, atherosclerosis, inflammation, and cancer. Molecular docking confirmed stable binding interactions between the five major active compounds—quercetin, apigenin, rosmarinic acid, salvigenin, and cirsimaritin—and the five core targets (AKT1, TP53, TNF, IL6, MAPK1). Among them, quercetin exhibited the strongest binding affinity with AKT1. RT-qPCR validation in clinical DFU tissue samples demonstrated consistent expression trends with computational predictions: AKT1 was significantly upregulated, while TP53, TNF, IL6, and MAPK1 were markedly downregulated in the Fespixon-treated group compared to controls (*p* < 0.001), supporting the proposed multi-target therapeutic mechanism. **Conclusions:** Our study reveals the potential mechanisms by which Fespixon cream exerts therapeutic effects on DFUs. The efficacy of Fespixon cream in treating DFUs is attributed to the synergistic actions of its bioactive components through multiple targets and multiple signaling pathways.

## 1. Introduction

Diabetic foot ulcers (DFUs) are a serious and prevalent complication of diabetes mellitus, with a lifetime incidence of up to 25% among diabetic patients and representing one of the leading causes of non-traumatic lower limb amputation worldwide [1,2]. The chronic nature of DFUs arises from a multifactorial pathogenesis involving peripheral neuropathy, ischemia, infection, and impaired wound healing. Recent research has elucidated that excessive oxidative stress, persistent low-grade inflammation, impaired angiogenesis, and dysfunction in macrophage polarization are major contributors to delayed healing in diabetic wounds [3,4]. These pathological processes are intricately linked to the diabetic microenvironment, where hyperglycemia induces reactive oxygen species (ROS) accumulation, promotes the activation of nuclear factor-κB (NF-κB), and upregulates pro-inflammatory cytokines such as TNF-α and IL-6, thereby perpetuating a vicious cycle of inflammation and tissue degradation. Furthermore, insufficient vascularization and reduced nitric oxide bioavailability compromise oxygen delivery, resulting in a hypoxic wound bed that impairs fibroblast function and keratinocyte migration [5]. Standard clinical care, including debridement, offloading, infection control, and glycemic management, while essential, is often insufficient for achieving full re-epithelialization, particularly in chronic or recurrent DFUs. Therefore, adjuvant therapies that target the underlying microenvironmental dysregulation have become an area of growing interest. Recent guidelines and meta-analyses have emphasized the importance of regenerative therapies—including growth factor supplementation, stem cell-based approaches, and botanical agents—as adjunctive modalities capable of addressing the biological impediments to wound healing [6].

Fespixon cream (commercially known as ON101), a topical formulation based on Traditional Chinese Medicine (TCM), has shown promising clinical efficacy in accelerating DFU healing. It contains two standardized botanical extracts: *Plectranthus amboinicus* (PA-F4) (commercially known as Daoshouxiang) and *Centella asiatica* (S1) (commercially known as Jixuecao). PA-F4 exhibits potent anti-inflammatory activity by suppressing NLRP3 inflammasome-mediated cytokine release, while S1 promotes angiogenesis, fibroblast proliferation, and collagen synthesis [7,8,9]. A multicenter, randomized controlled phase III clinical trial reported that Fespixon cream significantly improved wound closure rates compared to standard treatment (60.7% vs. 35.1% within 16 weeks, *p* < 0.001) [7]. Moreover, emerging evidence suggests that its bioactive components modulate key signaling pathways involved in oxidative stress, macrophage polarization, and extracellular matrix remodeling—thus offering a multifaceted therapeutic advantage over monotherapeutic agents [7].

However, due to the multi-component and multi-target nature of herbal formulations, the precise molecular mechanisms underlying Fespixon cream’s efficacy remain incompletely understood. In this context, network pharmacology, which integrates computational tools to analyze interactions between active compounds and biological targets in a systems biology framework, provides a powerful approach to uncover the holistic mechanisms of complex formulations [10]. Furthermore, molecular docking techniques enable structural-level validation of compound–target interactions by predicting binding affinity and stability [11]. Recent studies have increasingly utilized molecular docking to validate interactions between botanical compounds and diabetic wound healing targets. For example, quercetin, apigenin, and rosmarinic acid have demonstrated strong binding affinities to targets such as AKT1, TNF-α, and IL-6 in both in silico and preclinical studies. These findings support the rationale for integrating molecular docking into the investigation of Fespixon’s mechanism of action [12,13,14].

Therefore, in this study, we utilized an integrated network pharmacology and molecular docking approach to systematically identify the active ingredients, potential therapeutic targets, and key signaling pathways of Fespixon cream in the treatment of diabetic foot ulcers. Importantly, we further validated the mRNA expression trends of these targets in wound edge tissue samples from DFU patients using RT-qPCR, demonstrating consistency with the computational predictions. This integrative strategy not only clarifies the pharmacological basis of Fespixon cream but also provides robust experimental evidence supporting its clinical potential in DFU therapy.

## 2. Methods

### 2.1. Component and Target Analysis of Fespixon Cream

The chemical constituents of *Centella asiatica* (Jixuecao) were retrieved from the TCMSP (https://www.tcmsp-e.com, accessed on 10 March 2025). For *Plectranthus amboinicus* (Daoshouxiang), which is not listed in TCMSP, its active compounds were identified through relevant published literature [9]. The corresponding molecule IDs (MolID) for each compound were obtained from the TCMSP database when available. Given the characteristics of transdermal drug delivery systems, the following pharmacokinetic parameters were applied as screening thresholds: drug-likeness (DL) ≥ 0.18, logarithmic partition coefficient (AlogP) between 1 and 4, and molecular weight (MW) < 500 Da.

DL reflects whether a compound possesses certain functional groups or physicochemical properties similar to those of known drugs.

AlogP represents the logarithm of the octanol–water partition coefficient, indicating the compound’s lipophilicity or hydrophilicity, which is a key parameter for skin permeability.

Compounds meeting these criteria were selected as potential active ingredients. Their predicted protein targets were retrieved, and each corresponding protein was then input into the UniProt Knowledgebase (UniProtKB, https://www.uniprot.org/, accessed on 10 March 2025) to obtain standardized gene names. The search was restricted to the organism *Homo sapiens* to ensure human relevance. The protein identifiers were then converted to their respective gene symbols for further analysis.

### 2.2. Target Identification in Diabetic Foot Ulcers and Therapeutic Potential of Fespixon Cream

The term “Diabetic Foot Wound” was used as the keyword to search for disease-related targets in two databases: the GeneCards Human Gene Database (https://www.genecards.org/, accessed on 10 March 2025) and the Online Mendelian Inheritance in Man (OMIM) database (https://www.omim.org/, accessed on 10 March 2025). All identified targets associated with diabetic foot ulcers were collected, merged, and duplicates removed to obtain a comprehensive list of DFU-related genes. These disease-related targets were then intersected with the predicted targets of the active compounds in Fespixon cream to identify potential therapeutic targets. The overlapping genes represent the putative targets through which Fespixon cream may exert therapeutic effects in the treatment of diabetic foot ulcers. To visualize the relationship between drug targets and disease targets, a Venn diagram was generated using an online Venn Diagram tool (https://bioinformatics.psb.ugent.be/webtools/Venn/, accessed on 10 March 2025). This allowed for intuitive presentation of shared and unique targets between the compound and disease datasets.

### 2.3. Network Construction and Analysis of Fespixon Cream Components and Targets

Information on medicinal herbs, active compounds, and overlapping targets was organized using Microsoft Excel to create two input files: a “Network” file and a “Type” attribute file. These files were then imported into Cytoscape version 3.7.2, a network visualization software platform. A compound–target interaction network was constructed to illustrate the relationships among Fespixon cream, its bioactive ingredients, and their associated therapeutic targets. In the network, herbs, compounds, and targets were represented as nodes, and their interactions as edges. Topological analysis of the network was conducted using the NetworkAnalyzer plugin in Cytoscape, which computed key parameters such as degree, betweenness centrality, and closeness centrality to identify the most influential nodes. Compounds with high degree values were considered key active ingredients, while targets with high centrality were considered core therapeutic targets.

### 2.4. Protein–Protein Interaction Mapping and Functional Analysis

The intersecting targets between Fespixon cream and diabetic foot ulcer (DFU) were imported into the STRING database (Search Tool for the Retrieval of Interacting Genes/Proteins; https://string-db.org/, accessed on 10 March 2025) to construct a protein–protein interaction (PPI) network. The organism was set to *Homo sapiens* (human), and the interaction type was limited to “experiments and databases” with a minimum required confidence score of 0.7 (high confidence). Unconnected proteins were hidden from the network. The resulting PPI network data were exported from STRING and subsequently imported into Cytoscape version 3.7.2 for visualization and further analysis. Network topology analysis was performed using the CytoNCA plugin version 2.1.6, which calculates centrality measures such as betweenness centrality, closeness centrality, and degree centrality. Based on these metrics, core targets within the PPI network were identified as potential key regulators of the therapeutic effect of Fespixon cream on DFUs.

### 2.5. GO and KEGG Pathway Enrichment Analysis

Gene Ontology (GO) and Kyoto Encyclopedia of Genes and Genomes (KEGG) pathway enrichment analyses were conducted on the predicted therapeutic targets of Fespixon for diabetic foot ulcers using R software (version 4.4.2). The following R packages from the Bioconductor project (BiocManager) were utilized: “DOSE”, “clusterProfiler”, “enrichplot”, and “enrichKEGG”. Targets were annotated into GO categories, including biological process (BP), molecular function (MF), and cellular component (CC). KEGG enrichment was used to identify signaling pathways significantly associated with the shared targets. A *p*-value threshold of <0.05 was set to define statistically significant enrichment. The results were visualized using the Bioinformatics online platform (http://www.bioinformatics.com.cn, accessed on 10 March 2025), which generated bar plots and bubble plots to display the top-ranked enriched GO terms and KEGG pathways.

### 2.6. Molecular Docking Validation

Molecular docking is a widely used computational technique in drug discovery that predicts the preferred binding orientation and interaction affinity between small-molecule ligands and target proteins with known three-dimensional (3D) structures. In this study, molecular docking was performed to further evaluate the binding stability and reliability of key bioactive components of Fespixon with core therapeutic targets related to diabetic foot ulcers. The two-dimensional (2D) structures of the selected ligands were obtained from the PubChem database (https://pubchem.ncbi.nlm.nih.gov/, accessed on 10 March 2025). These were converted into energy-minimized three-dimensional (3D) conformations using ChemBio3D Ultra version 14.0. Corresponding receptor proteins were identified using the UniProt database (https://www.uniprot.org/, accessed on 10 March 2025) and retrieved from the Protein Data Bank (PDB) (http://www.rcsb.org/pdb, accessed on 10 March 2025) based on human-specific protein IDs. Protein preparation steps included removal of water molecules and ligands, hydrogenation, and addition of non-polar hydrogens using PyMOL software (version 2.3.4). Polar hydrogens were added, and non-polar hydrogens were merged using AutoDockTools version 1.5.6. The docking grid was defined using the Grid Box tool in AutoDockTools, centering the box on the known active site or ligand binding pocket of each target. Grid dimensions were typically set to 40 × 40 × 40 Å^3^ with a grid spacing of 1.0 Å, adjusted as needed to fully cover the binding pocket. Molecular docking was then performed using AutoDock Vina version 1.1.2, a semi-flexible docking approach that treats the receptor as rigid while allowing full flexibility of the ligand. The exhaustiveness parameter was set to 8 to ensure sufficient sampling of conformational space, and the maximum number of binding modes was set to 10. The docking results were evaluated based on the binding affinity (expressed in kcal/mol), with lower binding energy indicating a more stable ligand–receptor interaction. Binding poses with the most favorable energies were visualized using PyMOL, highlighting key hydrogen bonds and hydrophobic interactions to interpret molecular recognition patterns.

To ensure the reliability of the docking results, all target protein structures were retrieved from the RCSB Protein Data Bank (PDB) and prioritized based on resolution and experimental method. Only crystal structures derived from X-ray diffraction with high resolution (typically < 2.5 Å) were selected, as they provide more accurate atomic coordinates for ligand-binding analysis. For targets lacking co-crystallized ligands, a blind docking strategy was employed by covering the entire protein surface to approximate the active binding site. The binding pose with the lowest binding energy was subsequently selected as the most probable binding site. Mutations present in certain PDB entries were not the basis for structure selection.

### 2.7. RT-qPCR Experiment

To further verify whether the expression of predicted biomarkers in diabetic foot ulcer (DFU) tissues was consistent with the results of bioinformatics analysis, we collected wound edge tissue samples from 15 DFU patients treated with Fespixon cream and 15 non-Fespixon cream treated DFU control patients at Capital Medical University Affiliated Beijing Shijitan Hospital. The relative mRNA expression levels of core genes were assessed using RT-qPCR. All participants provided written informed consent prior to enrollment, and the study protocol was approved by the Ethics Committee of Capital Medical University Affiliated Beijing Shijitan Hospital (Approval No. IIT2025-062-001). RNA was extracted from the samples using TRIzol reagent (Invitrogen, 15596018, Carlsbad, CA, USA); reverse transcription was performed using the Hifair^®^ III 1st Strand cDNA Synthesis SuperMix for qPCR kit (Yeasen Biotechnology, 11141ES60, Shanghai, China), and primers were synthesized by Shanghai Shengong Biotech Co., Ltd. (Shanghai, China). The expression levels of biomarkers were calculated using the 2^−ΔΔCt^ method from three independent replicates of RT-qPCR experiments with GAPDH as the reference gene, and the differences in biomarker expression between DFU and control samples were analyzed using Student’s *t*-test in GraphPad Prism 10 software (*p* < 0.05). The experimental reaction system, conditions, and related primer sequences are referred to in Table 1, Table 2, Table 3, Table 4 and Table 5.

### 2.8. Statistical Analysis

All statistical analyses were performed using R statistical software (v4.4.2). Differences between two groups were compared using the Wilcoxon rank-sum test (*p* < 0.05). In RT-qPCR analysis, statistical comparisons were made using the *t*-test (*p* < 0.05).

## 3. Results

### 3.1. Screening of Active Components and Related Targets of Fespixon Cream

A total of eight active compounds were identified from *Centella asiatica* and four compounds from *Plectranthus amboinicus*, based on the screening criteria of oral bioavailability (OB ≥ 30%), drug-likeness (DL ≥ 0.18), and molecular weight (MW < 500 Da). Each compound was associated with one or more potential protein targets, which were retrieved and annotated via the UniProt database (https://www.uniprot.org/, accessed on 10 March 2025), with filtering conditions set to “Reviewed” entries and species limited to *Homo sapiens* (human). After de-duplication, a total of 202 unique targets were identified as the predicted targets of Fespixon cream.

### 3.2. Identification of Diabetic Foot Ulcer-Related Targets and Prediction of Potential Therapeutic Targets of Fespixon Cream

**Figure 1:**
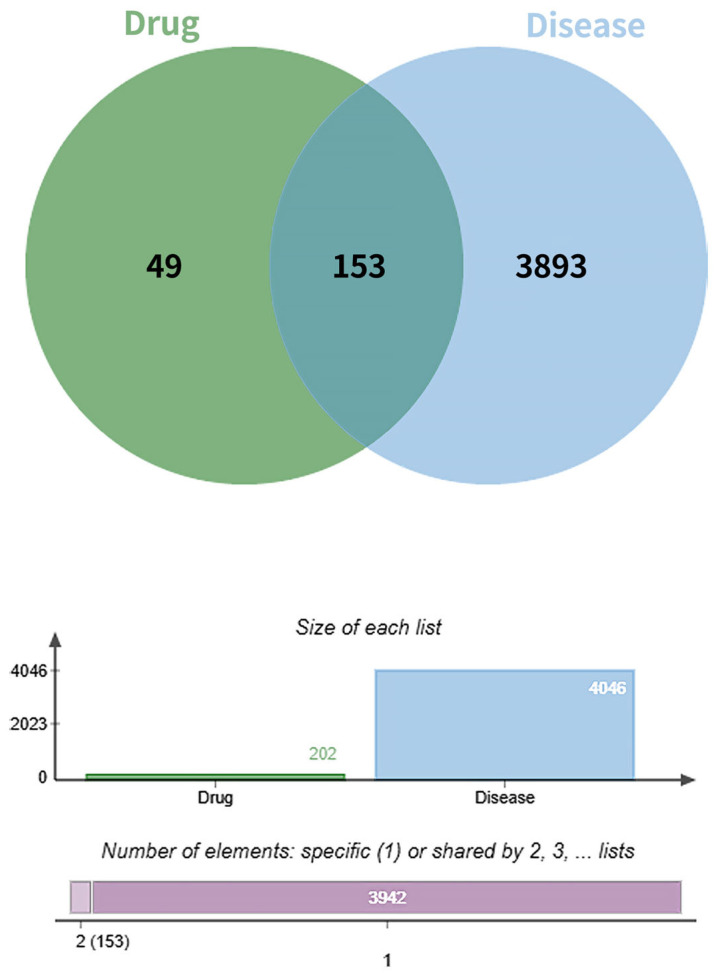
Venn diagram of the targets of Fespixon cream and diabetic foot ulcer-related targets.

A total of 4046 diabetic foot ulcer (DFU)-related targets were retrieved from the GeneCards and Online Mendelian Inheritance in Man (OMIM) databases by using the keyword “Diabetic Foot Wound”. After removal of duplicates, these targets were intersected with the 202 predicted targets of the active compounds in Fespixon. The intersection yielded 153 overlapping targets, which were considered potential therapeutic targets for Fespixon cream in the treatment of DFU (Figure 1).

### 3.3. Construction and Analysis of the Fespixon Cream–Compound–Target Network

**Figure 2:**
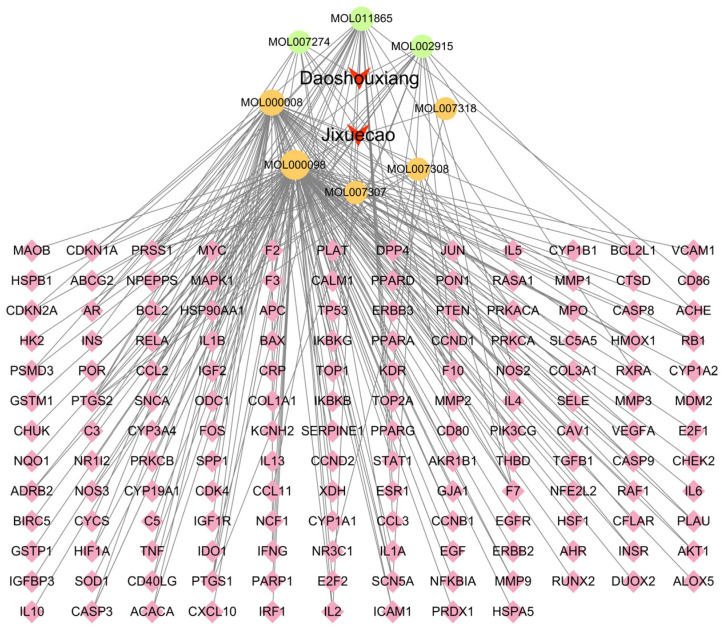
Network of Fespixon cream–compound–diabetic foot ulcer-related targets constructed using Cytoscape. Red nodes represent herbal medicines (e.g., Daoshouxiang, Jixuecao), Orange nodes represent bioactive compounds, Green nodes represent other ingredients, Pink diamond-shaped nodes represent diabetic foot ulcer-related target genes. Edges indicate the interactions between herbs, compounds, and targets.

The interactions between the active compounds of Fespixon cream and their corresponding diabetic foot ulcer (DFU)-related targets were visualized using Cytoscape version 3.7.2, resulting in a compound–target interaction network (Figure 2). The network consisted of 163 nodes (including herbs, compounds, and targets) and 254 edges, which represent the predicted interactions between the bioactive components and therapeutic targets. In the network diagram, squares represent herbal sources, circles represent active compounds, and diamonds denote potential therapeutic targets. Topological analysis based on the degree value identified the five most connected active compounds: quercetin, apigenin, rosmarinic acid, salvigenin, and cirsimaritin (Table 6). These high-degree nodes are considered key active constituents that may play principal pharmacological roles in the treatment of DFU by Fespixon cream.

### 3.4. Construction and Analysis of the Protein–Protein Interaction (PPI) Network

**Figure 3:**
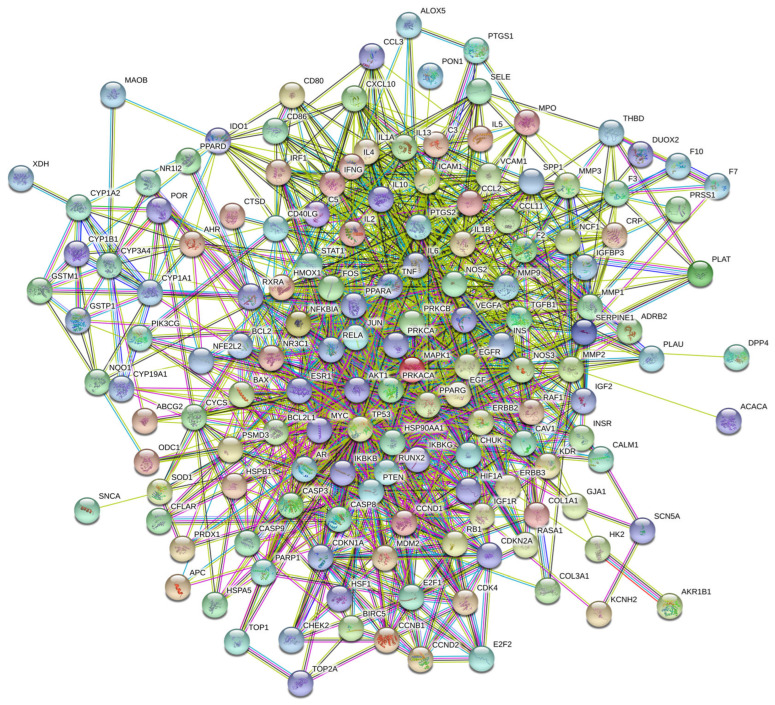
PPI network of potential therapeutic targets of Fespixon cream for the treatment of DFU.

**Figure 4:**
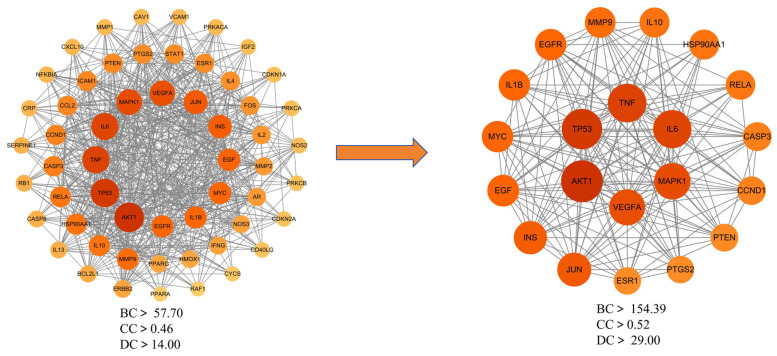
Core target subnetwork of Fespixon cream for diabetic foot ulcer treatment identified by topological analysis.

To explore the interactions among the 153 potential therapeutic targets of Fespixon cream for diabetic foot ulcers (DFUs), a protein–protein interaction (PPI) network was constructed using the STRING database (https://string-db.org/, accessed on 10 March 2025). The organism was set to *Homo sapiens*, and the minimum required interaction score was set to 0.7 (high confidence). Unconnected nodes were hidden, and all other parameters were set to default. The resulting PPI network consisted of 150 nodes and 1321 edges (Figure 3).The interaction data were then imported into Cytoscape version 3.7.2, where topological analysis was performed using the CytoNCA plugin. Based on the betweenness centrality (BC), closeness centrality (CC), and degree centrality (DC) values, a two-step screening process was applied to extract key nodes. As a result, 21 core targets were identified (Figure 4 and Table 7). Among these, the top five hub genes ranked by degree were: AKT1, TP53, TNF, IL6, and MAPK1. These genes occupied central positions in the network and are considered the major hub targets through which Fespixon cream may exert its therapeutic effects on DFUs.

### 3.5. GO Enrichment Analysis

**Figure 5:**
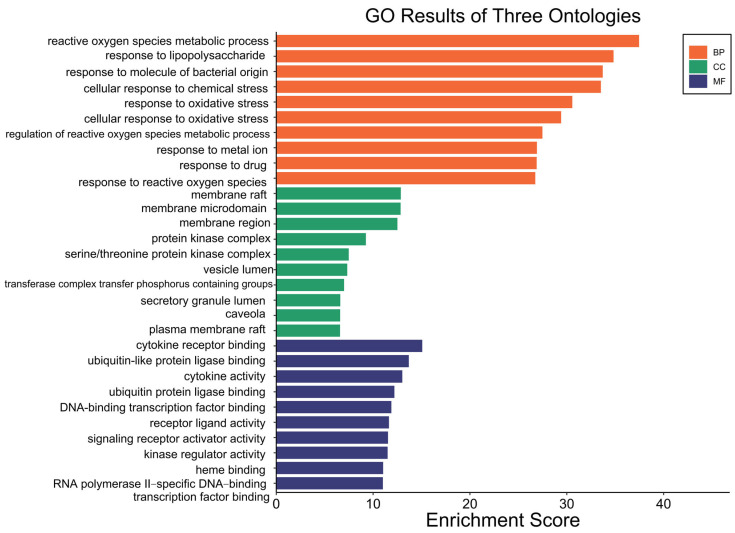
GO functional enrichment of potential therapeutic targets of Fespixon cream for the treatment of diabetic foot ulcers.

Gene Ontology (GO) enrichment analysis was performed on the 153 overlapping targets of Fespixon cream using R software. The targets were categorized into three functional domains: biological process (BP), molecular function (MF), and cellular component (CC). A total of 2607 BP terms were significantly enriched, primarily associated with reactive oxygen species (ROS) metabolic processes, response to lipopolysaccharide, response to molecules of bacterial origin, cellular response to chemical stress, and response to oxidative stress. For molecular function (MF), 200 enriched terms were identified, involving functions such as cytokine receptor binding, ubiquitin-like protein ligase binding, regulation of cytokine activity, and DNA-binding transcription factor activity. Regarding cellular component (CC), 73 enriched terms were found, which were mainly related to membrane rafts, membrane microdomains, protein kinase complexes, and vesicle lumens. The top 10 enriched GO terms across BP, MF, and CC categories were visualized in a bar plot using the Bioinformatics online platform (http://www.bioinformatics.com.cn, accessed on 10 March 2025) (Figure 5).

### 3.6. KEGG Pathway Enrichment Analysis

**Figure 6:**
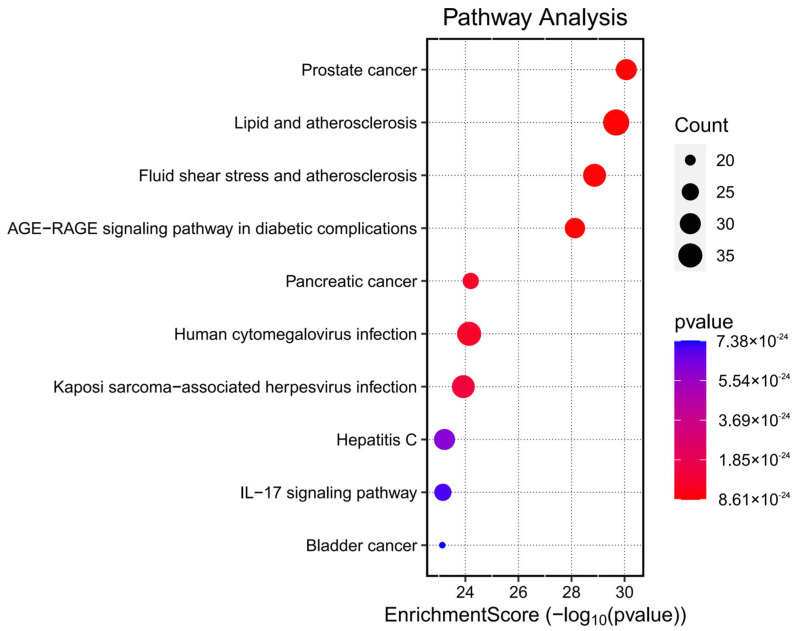
Bubble chart of KEGG pathway enrichment analysis for the potential therapeutic targets of Fespixon cream in the treatment of diabetic foot ulcers.

Kyoto Encyclopedia of Genes and Genomes (KEGG) pathway enrichment analysis was conducted on the 153 potential therapeutic targets of Fespixon cream using the “enrichKEGG” function from the clusterProfiler R package, along with the “pathview” library for visualization. A total of 185 KEGG pathways were significantly enriched (*p* < 0.05). These pathways were primarily involved in several biological processes related to inflammation, vascular dysfunction, and tumor signaling, including the prostate cancer pathway, lipid and atherosclerosis pathway, fluid shear stress and atherosclerosis, AGE-RAGE signaling pathway in diabetic complications, and the pancreatic cancer pathway. The top 10 enriched pathways ranked by *p*-value were visualized using a bubble chart (Figure 6), and detailed enrichment data are summarized in Table 8.

### 3.7. Molecular Docking Validation Between Key Targets and Active Compounds

Binding energy is considered a critical parameter for evaluating the stability and affinity of interactions between small-molecule ligands and protein targets. In this study, molecular docking was performed to assess the binding interactions between five core protein targets—AKT1, TP53, TNF, IL6, and MAPK1—and the top five bioactive compounds of Fespixon cream: quercetin, apigenin, rosmarinic acid, salvigenin, and cirsimaritin. The ligands quercetin, apigenin, rosmarinic acid, salvigenin, and cirsimaritin were optimized to their minimum energy state prior to docking. The 2D structures were retrieved from the PubChem database and converted to 3D conformations using ChemBio3D Ultra 14.0. The MM2 force field was applied to minimize energy and optimize molecular geometry to ensure physiochemical relevance during docking.

**Figure 7:**
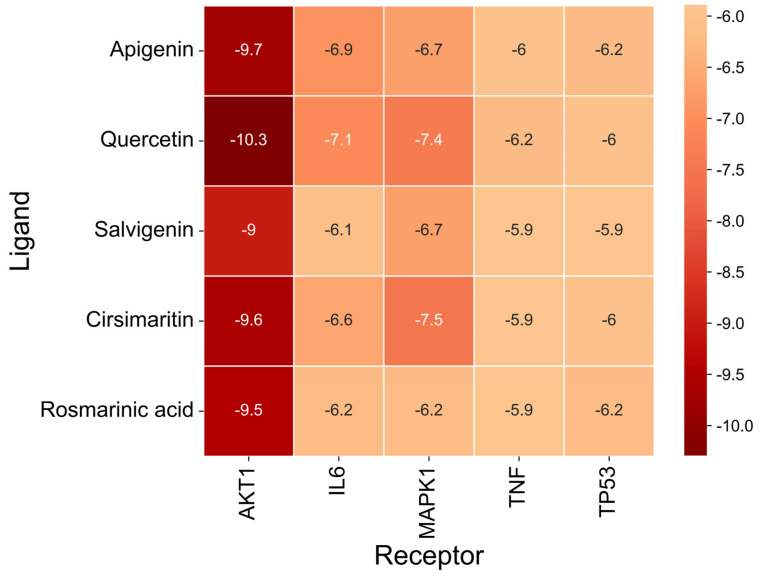
Heatmap of binding energies from molecular docking between core DFU-related targets and major active compounds of Fespixon cream.

**Figure 8:**
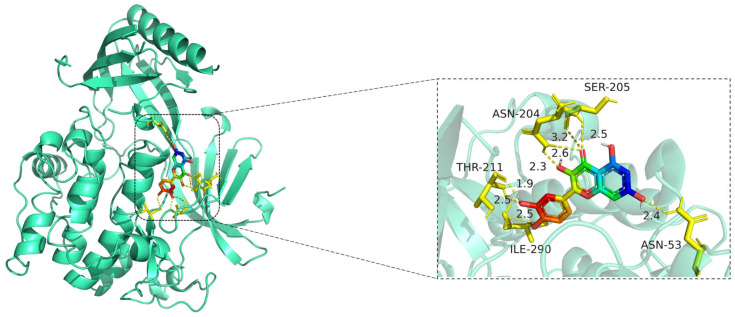
Molecular docking model of quercetin bound to AKT1. **Left**: Overall protein–ligand complex (AKT1 in cartoon representation; quercetin in stick representation). **Right**: Enlarged view of the binding interface. Hydrogen bonds (yellow dashed lines) are formed between quercetin and key residues including ASN-204, SER-205, THR-211, ILE-290, and ASN-53, with distances indicated in angstroms (Å). The protein AKT1 is shown in cartoon representation (cyan). The ligand quercetin is displayed in stick representation, with atoms colored by element: carbon (green), oxygen (red), and nitrogen (blue). Key interacting amino acid residues are shown in yellow stick form, and hydrogen bonds between quercetin and residues (ASN-204, SER-205, THR-211, ILE-290, and ASN-53) are indicated by yellow dashed lines, with corresponding bond distances labeled in angstroms (Å).

**Figure 9:**
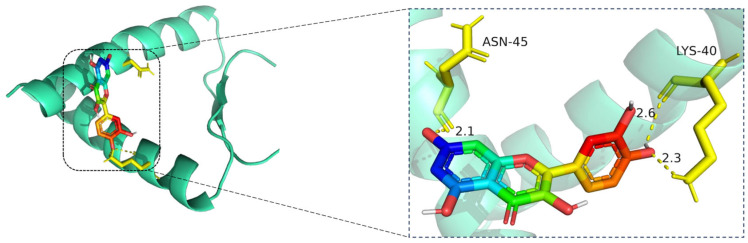
Molecular docking model of quercetin bound to TP53. **Left**: Overall view of the protein–ligand complex (TP53 shown as a cartoon; quercetin in stick representation). **Right**: Enlarged view of the binding interface. Hydrogen bonds (yellow dashed lines) are observed between quercetin and critical residues including ASN-45 and LYS-40, with bonding distances of 2.1 Å, 2.3 Å, and 2.6 Å indicated. These interactions contribute to the stability of quercetin within the binding pocket. The TP53 protein is shown in cartoon representation (cyan), with quercetin displayed in stick format, where carbon atoms are colored green, oxygen red, and nitrogen blue. Key interacting residues (ASN-45 and LYS-40) are rendered in yellow stick representation. Hydrogen bonds between quercetin and the binding site residues are indicated by yellow dashed lines, with bond distances labeled in angstroms (Å).

**Figure 10:**
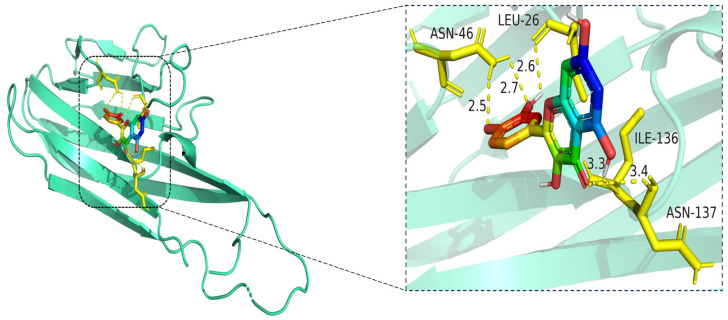
Molecular docking model of quercetin bound to TNF. **Left**: Overall view of the protein–ligand complex (TNF shown in cartoon representation; quercetin in stick representation). **Right**: Enlarged view of the binding interface. Hydrogen bonds (yellow dashed lines) are observed between quercetin and amino acid residues ASN-46, LEU-26, ILE-136, and ASN-137, with bond distances ranging from 2.5 Å to 3.4 Å. These interactions contribute to the stabilization of quercetin within the TNF binding pocket, indicating favorable binding affinity. The TNF protein is shown in cartoon representation (cyan), and the ligand quercetin is depicted in stick format, with atoms colored by element: carbon (green), oxygen (red), and nitrogen (blue). Key interacting residues (LEU-26, ASN-46, ILE-136, and ASN-137) are shown in yellow stick representation. Hydrogen bonds between quercetin and the binding site residues are illustrated as yellow dashed lines, with interaction distances labeled in angstroms (Å).

**Figure 11:**
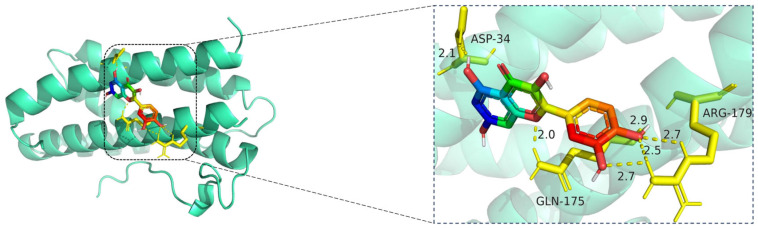
Molecular docking model of quercetin bound to IL6. **Left**: Overall view of the protein–ligand complex (IL6 shown in cartoon representation; quercetin in stick representation). **Right**: Enlarged view of the binding interface. Hydrogen bonds (yellow dashed lines) are formed between quercetin and key amino acid residues ASP-34, GLN-175, and ARG-179, with bond lengths ranging from 2.0 Å to 2.9 Å. These polar interactions help anchor quercetin in the IL6 binding site, suggesting potential inhibitory activity on IL6-mediated inflammatory signaling. The IL6 protein is displayed in cartoon representation (cyan), and the ligand quercetin is shown in stick format, with carbon atoms colored green, oxygen red, and nitrogen blue. Key amino acid residues involved in binding—ASP-34, GLN-175, and ARG-179—are represented in yellow sticks. Hydrogen bonds between quercetin and the binding pocket residues are illustrated with yellow dashed lines, and the bond distances are labeled in angstroms (Å).

**Figure 12:**
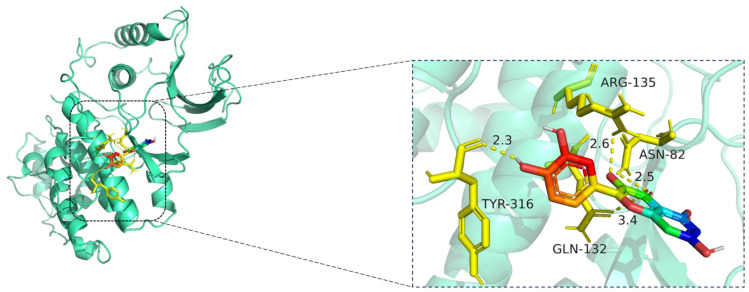
Molecular docking model of quercetin bound to MAPK1. **Left**: Overall view of the protein–ligand complex (MAPK1 shown in cartoon representation; quercetin in stick representation). **Right**: Enlarged view of the binding interface. Hydrogen bonds (yellow dashed lines) are observed between quercetin and amino acid residues TYR-316, GLN-132, ASN-82, and ARG-135, with bond distances ranging from 2.3 Å to 3.4 Å. These interactions indicate strong electrostatic stabilization and suggest that quercetin may modulate MAPK1 activity through key binding site engagement. The MAPK1 protein is shown in cartoon representation (cyan), while the ligand quercetin is displayed in stick format, with carbon atoms in green, oxygen in red, and nitrogen in blue. Key interacting residues—ASN-82, GLN-132, ARG-135, and TYR-316—are shown in yellow stick representation. Hydrogen bonds between quercetin and MAPK1 are illustrated as yellow dashed lines, with bond distances labeled in angstroms (Å).

The docking results (Figure 7 and Table 9) showed that all compound–target pairs exhibited minimum binding energies lower than −5.0 kcal/mol, suggesting favorable and stable interactions. Among them, quercetin showed the strongest binding affinity with AKT1, with a minimum binding energy of −10.3 kcal/mol, indicating that this compound–target interaction may play a central role in the pharmacological mechanism of Fespixon cream for diabetic foot ulcer treatment. The binding conformations were visualized using PyMOL software (Figure 8). Furthermore, the most potent active compound identified in our study—quercetin—was individually docked with the remaining four top-ranked core targets identified through PPI network construction and hub gene screening: TP53, TNF, IL6, and MAPK1. The corresponding binding conformations were also visualized using PyMOL (Figure 9, Figure 10, Figure 11 and Figure 12).

The docking visualization shows that quercetin fits well into the ATP-binding pocket of AKT1 and is stabilized by a network of hydrogen bonds and polar interactions involving key amino acid residues (Figure 8):

ASN-204 forms two hydrogen bonds with quercetin at distances of 2.6 Å and 3.2 Å, stabilizing the chromone ring.

SER-205 forms a hydrogen bond at 2.5 Å, likely contributing to polar anchoring.

THR-211 exhibits strong dual interactions with distances of 1.9 Å and 2.3 Å, suggesting close spatial proximity and strong electrostatic contribution.

ILE-290 interacts via hydrogen bonding (2.5 Å) and likely hydrophobic stabilization with the aromatic rings of quercetin.

ASN-53 contributes a polar interaction at 2.4 Å, located at the periphery of the binding pocket.

These residues collectively form a polar and hydrophobic microenvironment, tightly stabilizing the ligand. Notably, THR-211 and ASN-204 are located in the AKT1 hinge region and may influence kinase activity regulation, suggesting that quercetin could act as a competitive or allosteric modulator. The hydrogen bond distances (1.9–3.2 Å) fall within the optimal interaction range, reinforcing the high-affinity binding observed in the docking score (−10.3 kcal/mol) [15].

The docking visualization shows that quercetin fits well into the binding pocket of TP53 and is stabilized by a set of hydrogen bonds involving key amino acid residues (Figure 9):

ASN-45 forms a strong hydrogen bond with the hydroxyl group of quercetin at a distance of 2.1 Å, contributing to anchoring the chromone scaffold.

LYS-40 establishes two hydrogen bonds with quercetin, at distances of 2.6 Å and 2.3 Å, respectively, indicating electrostatic interactions with both the phenolic and ketone moieties of the ligand.

These interactions create a polar environment that stabilizes the quercetin conformation within the TP53 pocket. The involved residues (ASN-45 and LYS-40) are located near the DNA-binding domain of TP53, implying a potential regulatory influence on its transcriptional activity. The hydrogen bond distances ranging from 2.1 to 2.6 Å fall within the favorable interaction range, suggesting high binding affinity consistent with the docking results.

The docking visualization shows that quercetin fits well into the binding pocket of TNF-α and is stabilized by multiple hydrogen bonds and van der Waals interactions involving key amino acid residues (Figure 10):

ASN-46 forms a hydrogen bond with the hydroxyl group of quercetin at a distance of 2.5 Å, contributing to anchoring the ligand within the hydrophilic region.

LEU-26 establishes two hydrogen bonds at 2.6 Å and 2.7 Å, likely stabilizing the planar aromatic structure of quercetin.

ILE-136 and ASN-137 contribute to hydrophobic and polar contacts, respectively, with distances of 3.3 Å and 3.4 Å, indicating weaker but spatially close interactions that help stabilize the complex.

Collectively, these interactions create a moderately hydrophilic binding environment, stabilizing quercetin within the TNF-α trimer interface. The interaction distances fall within the favorable range for hydrogen bonding (2.5–3.4 Å), supporting the predicted binding affinity.

The docking visualization reveals that quercetin binds favorably to the IL6 receptor interface, stabilized by multiple hydrogen bonds with key residues (Figure 11):

ASP-34 forms a hydrogen bond at 2.1 Å with the hydroxyl group of quercetin, providing polar anchoring at the entrance of the binding pocket.

GLN-175 contributes significantly to ligand stabilization via three hydrogen bonds at 2.0 Å, 2.5 Å, and 2.7 Å, indicating strong spatial complementarity and charge-based interactions.

ARG-179 forms two hydrogen bonds at 2.7 Å and 2.9 Å with the chromone ring and phenolic moiety, reinforcing the electrostatic stability of the complex.

Together, these residues form a dense hydrogen bonding network, suggesting that quercetin has a strong and specific interaction with IL6. The bond lengths (ranging from 2.0 to 2.9 Å) fall well within optimal hydrogen bonding distances.

The docking visualization shows that quercetin fits stably within the ATP-binding site of MAPK1 and is anchored through several key hydrogen bonds and polar interactions (Figure 12):

TYR-316 forms a hydrogen bond with the hydroxyl group of quercetin at a distance of 2.3 Å, stabilizing the phenolic moiety.

ASN-82 interacts via a hydrogen bond at 2.5 Å, while ARG-135 forms another hydrogen bond at 2.6 Å, both contributing to polar stabilization within the binding pocket.

GLN-132 is positioned at 3.4 Å, suggesting weaker polar interactions with the chromone core of quercetin.

Together, these residues generate a polar microenvironment at the MAPK1 catalytic site, enhancing ligand binding. The interaction distances fall within optimal hydrogen bond range (2.3–3.4 Å), supporting the docking prediction.

### 3.8. Clinical Sample Experimental Validation

**Figure 13:**
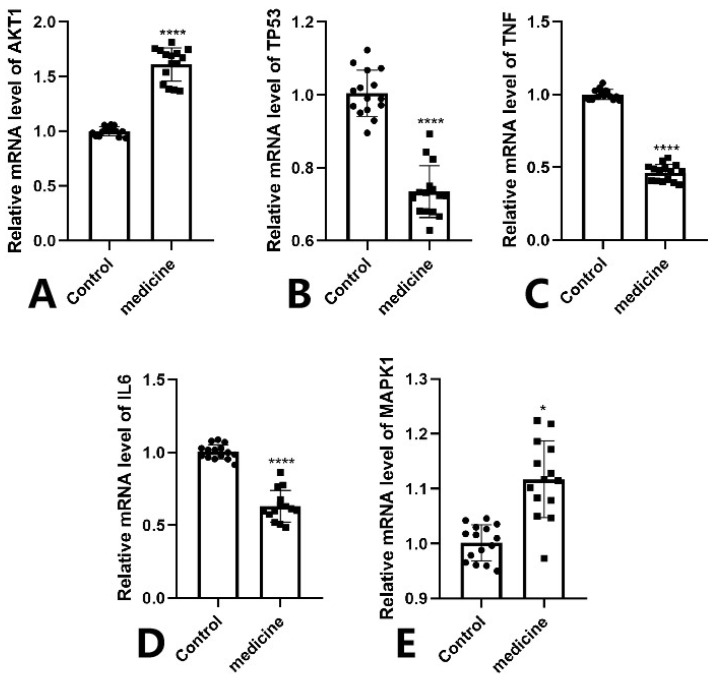
RT-qPCR validation of five core genes in wound tissue samples from DFU patients treated with Fespixon cream. (**A**) AKT1, (**B**) TP53, (**C**) TNF, (**D**) IL6, (**E**) MAPK1. Values are expressed as mean ± SD. **** *p* < 0.0001, * *p* < 0.05 vs. control.

The RT-qPCR results demonstrated that the mRNA expression levels of TP53,TNF and IL6 were significantly downregulated in the Fespixon cream-treated group compared to the control group (Figure 13B–D), while AKT1 was markedly upregulated (Figure 13A), and MAPK1 showed a mild but statistically significant increase (Figure 13E). These trends were consistent with the predictions from network pharmacology and molecular docking analyses. Statistical significance was confirmed as follows: *** *p* < 0.0001 for AKT1, IL6, TNF, and TP53; * *p* < 0.05 for MAPK1.

## 4. Discussion

Diabetic foot ulcers (DFUs) are chronic, non-healing wounds that result from a complex interplay of metabolic and vascular dysfunction. Persistent hyperglycemia induces excess oxidative stress and chronic inflammation, which impair normal wound healing processes [16]. Key mechanisms include: prolonged low-grade inflammation due to immune dysregulation; imbalance between ROS production and clearance; impaired angiogenesis and endothelial dysfunction [17].

Hyperglycemia-driven ROS accumulation promotes the overexpression of pro-inflammatory cytokines (e.g., TNF-α, IL-1β), matrix metalloproteinases (MMPs), and suppression of tissue inhibitors of metalloproteinases (TIMPs), ultimately resulting in extracellular matrix degradation [18,19]. Additionally, reduced nitric oxide bioavailability in endothelial cells inhibits neovascularization, leaving DFUs hypoxic and under-perfused. The wound microenvironment remains dominated by M1-type pro-inflammatory macrophages with inadequate transition to M2-type reparative macrophages, perpetuating tissue damage and impeding healing [19,20].

Conventional treatments often target isolated aspects of this complex pathology, resulting in suboptimal outcomes. Therefore, multi-target therapies—such as those offered by traditional botanical formulations—have gained attention for their potential to simultaneously address multiple pathological pathways. Recent evidence underscores the importance of multi-target therapeutic strategies in DFU. A *Panax notoginseng* saponin-based hydrogel delivering both antioxidant and pro-angiogenic factors was shown to simultaneously suppress oxidative stress and inflammatory senescence while promoting neovascularization, resulting in significantly accelerated wound closure in diabetic rats [21]. Likewise, dracorhodin (a flavonoid from Dragon’s Blood) improved DFU outcomes via multi-pathway modulation: it dose-dependently enhanced collagen deposition and angiogenesis while reducing inflammatory cytokines (TNF-α, IL-6) and ROS levels in a diabetic foot ulcer model [22]. These findings highlight that an integrative approach–dampening excessive inflammation and oxidative damage while boosting angiogenesis–yields markedly improved healing, supporting multi-target mechanism of action.

In this study, we applied an integrated network pharmacology and molecular docking approach, combined with RT-qPCR validation in clinical samples, to elucidate the mechanisms of action of Fespixon cream in treating DFUs. A total of 153 intersecting targets were identified between the active compounds of Fespixon cream and DFU-related genes, among which five hub targets—AKT1, TP53, TNF, IL6, and MAPK1—were found to play central roles in the interaction network. These targets are involved in critical processes including inflammation, oxidative stress regulation, cellular stress responses, and vascular regeneration. RT-qPCR analysis of wound edge tissues from DFU patients treated with Fespixon cream confirmed the regulatory trends predicted by in silico analysis: AKT1 was significantly upregulated, suggesting enhanced PI3K/Akt-mediated angiogenesis; TNF, IL6, and TP53 were markedly downregulated, reflecting attenuation of inflammation and stress-induced apoptosis; MAPK1 showed a mild but significant increase, indicating modulation of stress and repair signaling. These expression trends support the hypothesis that Fespixon cream reprograms the DFU microenvironment toward a pro-healing state. GO and KEGG enrichment analyses further demonstrated that Fespixon cream targets are significantly involved in biological pathways associated with AGE-RAGE signaling, HIF-1 and VEGF signaling, and lipid metabolism and atherosclerosis—all of which are implicated in DFU pathogenesis. The AGE-RAGE axis, in particular, is known to perpetuate chronic inflammation and vascular dysfunction under hyperglycemic conditions. Downregulation of RAGE-associated inflammatory targets such as TNF and IL6 in our study provides evidence of Fespixon cream’s regulatory effect on this pathway. The relationship between molecular docking predictions and experimental validation in this study highlights the multi-target therapeutic potential of Fespixon cream. Notably, quercetin exhibited the strongest binding affinity to AKT1 (−10.3 kcal/mol) and formed stable hydrogen bonds with catalytically relevant residues, which aligns with RT-qPCR results showing significant upregulation of AKT1 expression in DFU tissues after Fespixon treatment, suggesting activation of the PI3K/Akt pathway to promote angiogenesis. Similarly, TNF and IL6—predicted docking targets of apigenin and rosmarinic acid—were significantly downregulated in the experimental group, indicating suppression of inflammatory signaling. TP53, associated with oxidative stress, showed favorable binding and was also downregulated, while MAPK1 exhibited mild upregulation, consistent with its dual roles in stress response and tissue repair. These findings demonstrate that the transcriptional regulation observed in clinical samples reflects the predicted ligand–target interactions, reinforcing the pharmacological relevance of the docking results and supporting a synergistic, multi-target mechanism of Fespixon cream in diabetic wound healing.

The identification of AKT1, IL6, TP53, MAPK1, and TNF as Fespixon’s core targets is corroborated by recent mechanistic discoveries in the DFU pathology. Emerging studies confirm each of these molecules as pivotal nodes in wound-healing networks. AKT1 is a central pro-survival and pro-angiogenic kinase; activating AKT1 can promote angiogenesis and tissue repair. For instance, the natural iridoid gentiopicroside was found to accelerate diabetic ulcer healing by targeting AKT1 and up-regulating the HIF-1α/VEGF signaling axis [23]. Conversely, chronic hyperglycemia dysregulates the MAPK/ERK pathway (in which MAPK1 is a key component), impairing cellular proliferation and regeneration [24]. Restoring ERK signaling in diabetic wounds (e.g., via Wnt or growth factor therapies) is now recognized as a promising strategy to improve healing [24]. In parallel, excessive inflammation mediated by IL-6 and TNF-α remains a well-known barrier to DFU closure; elevated levels of these cytokines in non-healing ulcers lead to continual macrophage M1 polarization and inhibited keratinocyte migration [22]. Therapeutic interventions that lower IL-6 and TNF-α (while boosting growth factors) demonstrably expedite wound closure, underscoring their role as high-value targets [22]. Meanwhile, new insight into TP53 (p53) reveals that sustained p53 activation in diabetic wounds is deleterious: unlike acute wounds where p53 transiently declines, DFUs show prolonged p53 activity that enforces cell-cycle arrest and fuels chronic inflammation. A 2024 study found that hyperactivated p53 in diabetic ulcers drives cellular senescence and triggers inflammatory cell death (via cGAS-STING–NFκB signaling, ferroptosis and pyroptosis), thereby impeding tissue repair [25]. Such findings emphasize that moderating p53 activity (to relieve senescence and inflammation) can remove a significant roadblock to healing. In summary, the network-detected hubs AKT1, IL6, TP53, MAPK1, and TNF are not only theoretical targets but indeed proven key regulators of wound healing. Restoring the balance of these factors (e.g., by suppressing IL-6/TNF/p53-driven inflammation and invigorating AKT/MAPK pro-regenerative signaling) is a validated therapeutic paradigm in DFU management.

Our study findings are consistent with prior reports on the pharmacological activities of Fespixon’s core bioactive ingredients. For instance, quercetin has been shown to inhibit M1 macrophage polarization, promote VEGF expression via PI3K/Akt, and reduce ROS production. Apigenin modulates the miR-21/TLR/NF-κB axis, suppressing pro-inflammatory cytokines and promoting tissue repair. Rosmarinic acid exhibits strong antioxidant and anti-inflammatory effects, contributing to wound healing. Our molecular docking results confirmed strong binding affinities between these compounds and their respective protein targets, reinforcing their mechanistic plausibility. Growing animal and clinical evidence further attests to the efficacy of botanical and traditional medicine-based treatments in DFU, aligning with Fespixon’s multi-component strategy. In preclinical models, numerous natural compounds have shown potent wound-healing effects. Studies report that quercetin—a flavonoid present in many medicinal plants, and one of Fespixon’s active compounds—significantly accelerates diabetic wound closure by modulating multiple targets. Quercetin’s anti-inflammatory, anti-oxidant, and pro-angiogenic actions include shifting macrophages from the pro-inflammatory M1 state to the pro-healing M2 phenotype, thereby lowering TNF-α/IL-6 levels and boosting angiogenic factors in the wound bed [26]. This multi-faceted mechanism mirrors Fespixon’s intended mode of action and was confirmed in diabetic rat wounds treated with quercetin, which exhibited faster contraction, greater collagen deposition, and improved microvessel density compared to controls [26]. Crucially, botanical therapies have also translated into clinical success. ON101 (Fespixon^®^ cream) itself recently demonstrated superior efficacy in a Phase III multicenter trial: 60.7% of patients achieved complete DFU healing within 16 weeks with Fespixon, versus 35.1% in the standard care group (*p* = 0.0001) [7]. Likewise, other herbal formulations have shown promise in clinical or advanced preclinical studies, often by targeting the same triad of inflammation, oxidative stress, and impaired angiogenesis. For example, a traditional Chinese medicine compound (Jingfang granules) was reported to improve diabetic wound healing by inducing angiogenesis, reducing oxidative stress, and modulating inflammatory responses [27]. Taken together, these findings from animals and human patients validate that multi-component remedies—such as Fespixon—can effectively engage multiple molecular targets and pathways to overcome the multifactorial pathogenesis of DFU, thereby significantly enhancing wound healing outcomes. The convergence of network pharmacology predictions with experimental and clinical evidence strongly supports the mechanistic basis of Fespixon’s efficacy in treating diabetic foot ulcers [22].

Despite these promising results, several limitations should be acknowledged. First, the current findings are based primarily on computational predictions and expression-level validation; protein-level confirmation (e.g., via Western blot or immunohistochemistry) and functional assays (e.g., angiogenesis, macrophage polarization) are needed to establish causality. Second, the pharmacokinetics and transdermal bioavailability of the active compounds in Fespixon cream remain unclear. Third, the sample size in the clinical validation was limited, and further studies with larger cohorts and in vivo models are warranted.

Our study provides a comprehensive systems-level framework for understanding the multi-target therapeutic effects of Fespixon cream in DFU treatment. By modulating key regulators of inflammation, oxidative stress, and angiogenesis, Fespixon cream may effectively transform a hostile chronic wound environment into one conducive to repair. These results support the further development of Fespixon cream as a promising adjunct therapy for diabetic foot ulcers.

## 5. Conclusions

This study elucidates the potential multi-target therapeutic mechanisms of Fespixon cream in promoting diabetic foot ulcer (DFU) healing through an integrative approach combining network pharmacology, molecular docking, and experimental validation. Key active compounds such as quercetin, apigenin, and rosmarinic acid were shown to modulate core DFU-related targets including AKT1, TP53, TNF, IL6, and MAPK1, which are associated with inflammation, oxidative stress, and angiogenesis. RT-qPCR results in clinical wound edge tissues confirmed these predicted gene expression trends. Together, these findings suggest that Fespixon cream exerts its beneficial effects via a synergistic modulation of multiple pathological pathways, offering strong theoretical and experimental support for its clinical application in DFU treatment. Future studies should focus on in vivo functional validation and pharmacokinetic evaluation to further substantiate its translational potential.

## Figures and Tables

**Table 1:** cDNA reaction system.

Component	Volume
GoScript^TM^ Enzyme Mix	4 µL
GoScript^TM^ Reaction Buffer	4 µL
Total RNA	2 µg
Nuclease-Free Water	Add to 20 µL

**Table 2:** cDNA reaction conditions.

Temperature	Time
25 °C	5 min
42 °C	60 min
70 °C	15 min
4 °C	hold

**Table 3:** qPCR reaction system.

Component	Volume
cDNA	1 µL
2xUniversal Blue SYBR Green qPCR Master Mix	5 µL
Forward primer (10 µM)	0.25 µL
Reverse primer (10 µM)	0.25 µL
ddH_2_O	5.5 µL

**Table 4:** qPCR amplification conditions.

	Temperature	Time
Initial Denaturation	95 °C	5 min
Denaturation	95 °C	30 s
Annealing and Extension	60 °C	60 s

**Table 5:** Primer sequences.

Primer		Sequence
AKT1	F	AGAAGCAGGAGGAGGAGGAG
AKT1	R	CGACCGCACATCATCTCGTA
IL-6	F	ACCCCCAGGAGAAGATTCCA
IL-6	R	ATTTGTGGTTGGGTCAGGGG
TNF	F	TGTGGGGTGTGAGAAGAGAGA
TNF	R	GCTCTTAGCCCTGAGGTGTC
MAPK1	F	CAGTTCTTGACCCCTGGTCC
MAPK1	R	CTGGGACATCCCCAGAAACC
TP53	F	GGGTTGATTCCACACCCCC
TP53	R	CTCCGTCATGTGCTGTGACT
GAPDH	F	ATGGGCAGCCGTTAGGAAAG
GAPDH	R	AGGAAAAGCATCACCCGGAG

**Table 6:** Major active compounds of Fespixon cream.

ID	Molecule Name	Herb	Degree
MOL000098	Quercetin	*Centella asiatica* (Jixuecao)	126
MOL000008	Apigenin	*Centella asiatica* (Jixuecao)	59
MOL011865	Rosmarinic acid	*Plectranthus amboinicus* (Daoshouxiang)	26
MOL002915	Salvigenin	*Plectranthus amboinicus* (Daoshouxiang)	16
MOL007274	Cirsimaritin	*Plectranthus amboinicus* (Daoshouxiang)	10
MOL007308	Castilliferol	*Centella asiatica* (Jixuecao)	9
MOL007307	Castillicetin	*Centella asiatica* (Jixuecao)	6
MOL007318	Asiaticoside, f_qt	*Centella asiatica* (Jixuecao)	2

**Table 7:** Core potential therapeutic targets of Fespixon cream in the treatment of diabetic foot ulcers.

Gene	Betweenness	Closeness	Degree
*AKT1*	2429.38	0.642	73
*TP53*	2389.60	0.629	69
*TNF*	1293.53	0.616	64
*IL6*	1360.79	0.616	63
*MAPK1*	1237.83	0.598	58
*VEGFA*	1004.31	0.603	57
*JUN*	621.88	0.575	51
*INS*	1460.20	0.575	49
*EGF*	635.52	0.562	45
*EGFR*	520.63	0.558	44
*IL1B*	393.26	0.540	44
*MYC*	623.35	0.556	44
*MMP9*	674.79	0.556	42
*HSP90AA1*	928.77	0.542	40
*IL10*	264.42	0.546	40
*RELA*	276.21	0.538	39
*CASP3*	502.16	0.540	37
*CCND1*	418.66	0.528	36
*PTEN*	292.68	0.527	34
*ESR1*	332.27	0.536	33
*PTGS2*	378.29	0.528	33

**Table 8:** Top 10 enriched KEGG signaling pathways.

ID	Description	*p* Value	Count
hsa05215	Prostate cancer	8.61 × 10^−31^	30
hsa05417	Lipid and atherosclerosis	2.08 × 10^−30^	39
hsa05418	Fluid shear stress and atherosclerosis	1.35 × 10^−29^	33
hsa04933	AGE-RAGE signaling pathway in diabetic complications	7.43 × 10^−29^	29
hsa05212	Pancreatic cancer	6.27 × 10^−25^	24
hsa05163	Human cytomegalovirus infection	7.21 × 10^−25^	35
hsa05167	Kaposi sarcoma-associated herpesvirus infection	1.19 × 10^−24^	33
hsa05160	Hepatitis C	6.09 × 10^−24^	30
hsa04657	IL-17 signaling pathway	7.05 × 10^−24^	25
hsa05219	Bladder cancer	7.38 × 10^−24^	19

**Table 9:** Binding energies of molecular docking between core DFU-related targets and major active compounds of Fespixon cream.

Receptor	Uniport-ID	PDB-ID	Ligand	MOL-ID	Binding Energy (kcal·mol^−1^)
AKT1	P31749	1UNQ	Apigenin	MOL000008	−9.7
AKT1	P31749	1UNQ	Quercetin	MOL000098	−10.3
AKT1	P31749	1UNQ	Salvigenin	MOL002915	−9
AKT1	P31749	1UNQ	Cirsimaritin	MOL007274	−9.6
AKT1	P31749	1UNQ	Rosmarinic acid	MOL011865	−9.5
IL6	P05231	1ALU	Apigenin	MOL000008	−6.9
IL6	P05231	1ALU	Quercetin	MOL000098	−7.1
IL6	P05231	1ALU	Salvigenin	MOL002915	−6.1
IL6	P05231	1ALU	Cirsimaritin	MOL007274	−6.6
IL6	P05231	1ALU	Rosmarinic acid	MOL011865	−6.2
MAPK1	P28482	3SA0	Apigenin	MOL000008	−6.7
MAPK1	P28482	3SA0	Quercetin	MOL000098	−7.4
MAPK1	P28482	3SA0	Salvigenin	MOL002915	−6.7
MAPK1	P28482	3SA0	Cirsimaritin	MOL007274	−7.5
MAPK1	P28482	3SA0	Rosmarinic acid	MOL011865	−6.2
TNF	P01375	4TSV	Apigenin	MOL000008	−6
TNF	P01375	4TSV	Quercetin	MOL000098	−6.2
TNF	P01375	4TSV	Salvigenin	MOL002915	−5.9
TNF	P01375	4TSV	Cirsimaritin	MOL007274	−5.9
TNF	P01375	4TSV	Rosmarinic acid	MOL011865	−5.9
TP53	P04637	1AIE	Apigenin	MOL000008	−6.2
TP53	P04637	1AIE	Quercetin	MOL000098	−6
TP53	P04637	1AIE	Salvigenin	MOL002915	−5.9
TP53	P04637	1AIE	Cirsimaritin	MOL007274	−6
TP53	P04637	1AIE	Rosmarinic acid	MOL011865	−6.2

## Data Availability

Data is contained within the article.
